# Differentiating tics and functional tic-like behaviours in children and young people: personal and family mental health profiles and their clinical implications

**DOI:** 10.1007/s00787-026-03067-7

**Published:** 2026-05-26

**Authors:** Holan Liang, Savva Pronin, Morvwen Duncan, Lila Simpson, Abbie Smith, Katie Harrold, Zoe Pearman, Amy Warren, Sara Shavel-Jessop, Tara Murphy, Isobel Heyman, Sacha Evans, Matteo Catanzano

**Affiliations:** 1 Tic Service, Psychological and Mental Health Services, Great Ormond Street Hospital, London, UK; 2 University College London Great Ormond Street Institute of Child Health, London, UK; 3 Department of Psychiatry, Cambridge University, Cambridge, UK; 4 Northeast London Foundation Trust, London, UK; 5 Department of Child and Adolescent Psychiatry, Institute of Psychiatry, Psychology & Neuroscience, King’s College London, London, UK; 6 South London and Maudsley NHS Foundation Trust, London, UK; 7 The Tavistock and Portman NHS Foundation Trust, London, UK; 8 Cambridge and Peterborough NHS Foundation Trust, Cambridge, UK

**Keywords:** Functional tic-like behaviours, Attention-deficit hyperactivity disorder, Autism, Tourette’s syndrome, Demographics, Cross-sectional study

## Abstract

Aim. Personal and family mental health histories of youth with functional tic-like behaviours (FTLB) and/or tics (TS) were compared. Method. This is a cross-sectional study with between-group analysis of 127 patients aged 4–17 years referred to a tic service between 2019 and 2022 who were diagnosed with FTLB, TS, or co-occurring FTLB and TS. Co-occurring psychiatric and neurodevelopmental conditions in the personal and family history of cases collected prospectively were recorded as per standard care. Post-hoc analysis was undertaken to explore findings of interest. Results. Later tic onset (13 years vs. 5 years) and increased comorbid psychiatric disorders (> 78% vs. 51%) in the FTLB versus TS groups, support the European Society for the Study of Tourette Syndrome (ESSTS) criteria for clinical diagnosis of FTLBs. Anxiety, depression and autism were more prominent in FTLB groups. 35% of the FTLB group had an ADHD diagnosis. Family history of tics is significantly more likely in the TS groups, while family history of anxiety was most prominent in the TS+FTLB group. Post hoc analysis indicated that in FTLB with co-occurring ADHD, the majority of ADHD (56%) was picked up following FTLB presentation, with missed ADHD diagnoses mainly in girls. Discussion. Patterns of difference in mental health and family mental health exist between TS and FTLB supporting ESSTS diagnostic criterion for FTLB. FTLB is associated with anxiety, depression and autism. ADHD is present in 35% of FTLB even without TS, and if this is a replicable finding, suggests a treatable vulnerability factor in FTLB.

**Supplementary Information:**

The online version contains supplementary material available at https://doi.org/10.1007/s00787-026-03067-7.

## Introduction

Tics are sudden, rapid, non-rhythmic and repetitive movements or vocalisations, which can be seen in a variety of conditions, such as Tourette syndrome (TS) [1]. Tic-like movements or sounds can also occur as part of a functional neurological disorder (FND), which is an umbrella term also including functional seizures. Tics in this context are referred to as functional tic-like behaviours (FTLB). Since the COVID-19 pandemic, there has been growing concern surrounding the increase of FTLB, occurring predominantly in adolescents and young adults [2, 3], with rates growing from 2% in 2018 to 36% in 2021 within clinical samples [4]. This increase was particularly prominent in adolescent females [5] and was hypothesised to be associated with elevated rates of anxiety in vulnerable individuals. Pandemic related impacts, including the increase of social isolation, stress and anxiety [6], increased social media consumption during the pandemic [7] and the exposure to videos showcasing individuals with tics, may have been contributing factors in triggering FTLB [8].

Onset of FTLB is typically around the adolescent period compared to TS where onset tends to be in childhood [9]. Children who are diagnosed with FTLB are more likely to present with complex tics, including arm/hand tics, vocal tics and coprolalia, while those with neurodevelopmental tics tend to present with simple tics such as eye blinking [9]. However, differentiating FTLBs from neurodevelopmental tics can be challenging in clinical practice. Recent research suggested only ‘fair’ diagnostic agreement between expert assessors when using phenomenology alone [10]. To complicate diagnosis further, it is also possible to have a combination of tics and FTLB. Indeed, the literature suggests that 10–50% of functional seizures occur in young people with epilepsy [11], therefore we would expect a proportion of FTLB to occur in young people with tics.

Individuals with FTLB may often find their tics distressing, disruptive to their daily life and they can hinder social functioning [12]. Making an accurate diagnosis is important in this group because FTLBs tend not to respond to medication typically used for chronic tic disorder [13]. There is currently limited evidence on effective treatments that can be used to support the management of FTLB. Although young people face a better prognosis compared to adults, time is required for symptoms to improve [14]. Preliminary findings have suggested psychoeducation and Cognitive Behavioural Therapy (CBT) are useful in improving underlying anxiety and depression symptoms, and to aid young people in diverting unwanted attention away from tics [14, 15]. Nonetheless, more research is required on the underlying causes of FTLB to inform interventions.

To aid differentiation between TS and FTLB, The European Society for the Study of Tourette Syndrome (ESSTS) proposed three major criteria and two minor criteria for the clinical diagnosis of FTLBs. Major criteria include age at symptom onset after 12 years, a rapid evolution of symptoms (typically over hours to days) and at least 4 of 9 phenomenological features (which include larger number of motor and vocal complex tic-like movements, lack of rostrocaudal progression of tic appearance, large variation of symptom frequency, rapidly changing tics with variable reproduction). Minor criteria include comorbid psychiatric symptoms/diagnoses and a history of other functional symptoms. The criteria stipulate that a clinically definite diagnosis of FTLBs can be confirmed by the presence of all three major criteria, whereas a clinically probable diagnosis of FTLBs can be confirmed by the presence of two major criteria and one minor criterion [16].

ESSTS Minor Criterion 1 for FTLB: *‘Comorbid psychiatric symptoms and diagnoses’* include *‘predominance of preceding anxiety and depressive disorders (often undiagnosed and untreated)*,* self-harm behaviours (i.e.*,* cutting)*,* suicidal ideation*,* preceding traumatic and stressful life events*,* and dysfunctional family relationships. Social and school difficulties with friends and/or academics are often present.’* Such criteria are underpinned by data from the largest FTLB registry (*N* = 294), which revealed 80% of FTLB cases had a psychiatric comorbidity [17]. This is higher than the 39% reported rate of co-occurring psychiatric morbidity in youth with functional seizures [18]. Anxiety (66%) and depressive disorders (28%) were most common, while autism (24%) and attention deficit/hyperactivity disorder (ADHD) (23%) were the most common neurodevelopmental co-occurring conditions. Other comorbidities included a primary tic disorder (19%), post-traumatic stress disorder (PTSD) (9%) and other functional neurological symptoms (37%) including functional seizures (19%) [17]. Understandably, adults (who have lived longer to accumulate additional mental health difficulties) were more frequently reporting other FND symptoms and prior depressive disorder diagnoses than adolescents [17].

FTLB literature has reported statistical comparisons to TS controls indicating FTLB and FTLB + TS cases have higher rates of depression and anxiety [4, 13]. One study has reported on family history as a potential differentiating factor between TS and FTLB. In the absence of genetic studies, family history can give insight into whether genetic contribution is relevant to aetiology. For FTLB there tends to be an absence of family history of tic disorders and increased psychiatric family history [19].

Our study aims to replicate existing findings as the FTLB literature base remains small, with few studies of small sample size; therefore, replication within differing populations builds better clinical certainty of findings. In addition, we add 3 comparator groups in our sample which is unique. We compare children and young people with a diagnosis of chronic tic disorder/ Tourette Syndrome (TS group), FTLB (FTLB group) or co-occurring tics and FTLB (FTLB + TS group) with each other in terms of demographics, mental health co-morbidity profile and family history of mental health problems. Where findings are unexpected, we performed additional post-hoc analysis to attempt to understand the findings.

## Methods

### Design

This was a cross-sectional study using a prospectively collected cohort from a service audit of FTLB, including some cases which contributed to international FTLB datasets [17].

### Setting and participants

Recruitment of participants was sequential. Participants were young people aged 4–17 years referred to the Great Ormond Street Hospital (GOSH) Tic Service, a national and specialist service, between July 2019 and May 2022. Inclusion criteria included: under age 18, and a diagnosis of FTLB and/or TS following assessment. As the dataset was collected from a functioning clinic rather than based on research protocol, care offered differed depending on individual clinical circumstances, however during assessment, all participants underwent a clinical diagnostic assessment by the multidisciplinary team consisting of a consultant psychiatrist/ neurologist/ psychologist, clinical psychologists and resident doctors in psychiatry and neurology, the details of which are described elsewhere [20]. Primary diagnosis, other psychiatric and neurodevelopmental diagnoses, and family history were recorded at this point. Follow-up was pragmatic within July 2019 to May 2022 with any new mental health diagnosis recorded if/ when a young person re-attended the tic clinic for appointments.

### Main outcome measures

Information was collected from electronic health records and clinic letters for all patients, including: demographic information (e.g. age at initial assessment, sex assigned at birth, gender identity at assessment, ethnicity), age when tics first started, co-occurring psychiatric (e.g. depressive disorders, anxiety disorders, post-traumatic stress disorder, eating disorders, obsessive–compulsive disorder (OCD), oppositional defiant disorder) and neurodevelopmental (e.g. ADHD, autism, specific learning disorder (SpLD)) diagnoses. To determine family history, the reported neurodevelopmental and psychiatric diagnoses of first- and second-degree relatives were collected from the health record for biological relatives only. The disorders provided by the family were volunteered at clinical assessment and were not independently verified.

### Statistical analyses

Descriptive statistics were computed as appropriate with means (standard deviations), median (interquartile ranges) and percentages. Two-way comparisons between children in FTLB and TS and TS+FTLB groups were carried out using chi-square/Fisher’s exact test, or independent t-test/Mann Whitney U tests, as appropriate. As the analyses were exploratory, we did not correct for multiple comparisons. All statistical analyses were carried out using RStudio (version 4.1.2., R Foundation for Statistical Computing, Vienna, Austria).

### Ethics statement

Approval was granted by the clinical audit department at GOSH (audit reference number: 2420). Although collection and analysis of data did not require ethical review by an NHS research ethics committee, care was taken to ensure ethical standards were met. No personally identifiable data was detailed.

## Results

### Demographic variables

The cohort consisted of 127 participants; 50% (*n* = 63) had FTLB, 69% (*n* = 87) had TS, and 18% (*n* = 23) had both FTLB and TS. During this period 68% of patients seen for ‘tics’ involved FTLB. Demographics of the total cohort were: 54% (n = 68) assigned female at birth; mean age 13 years (SD 2.7); and 60% (n = 76) identified as ‘white’.

**Fig. 1:**
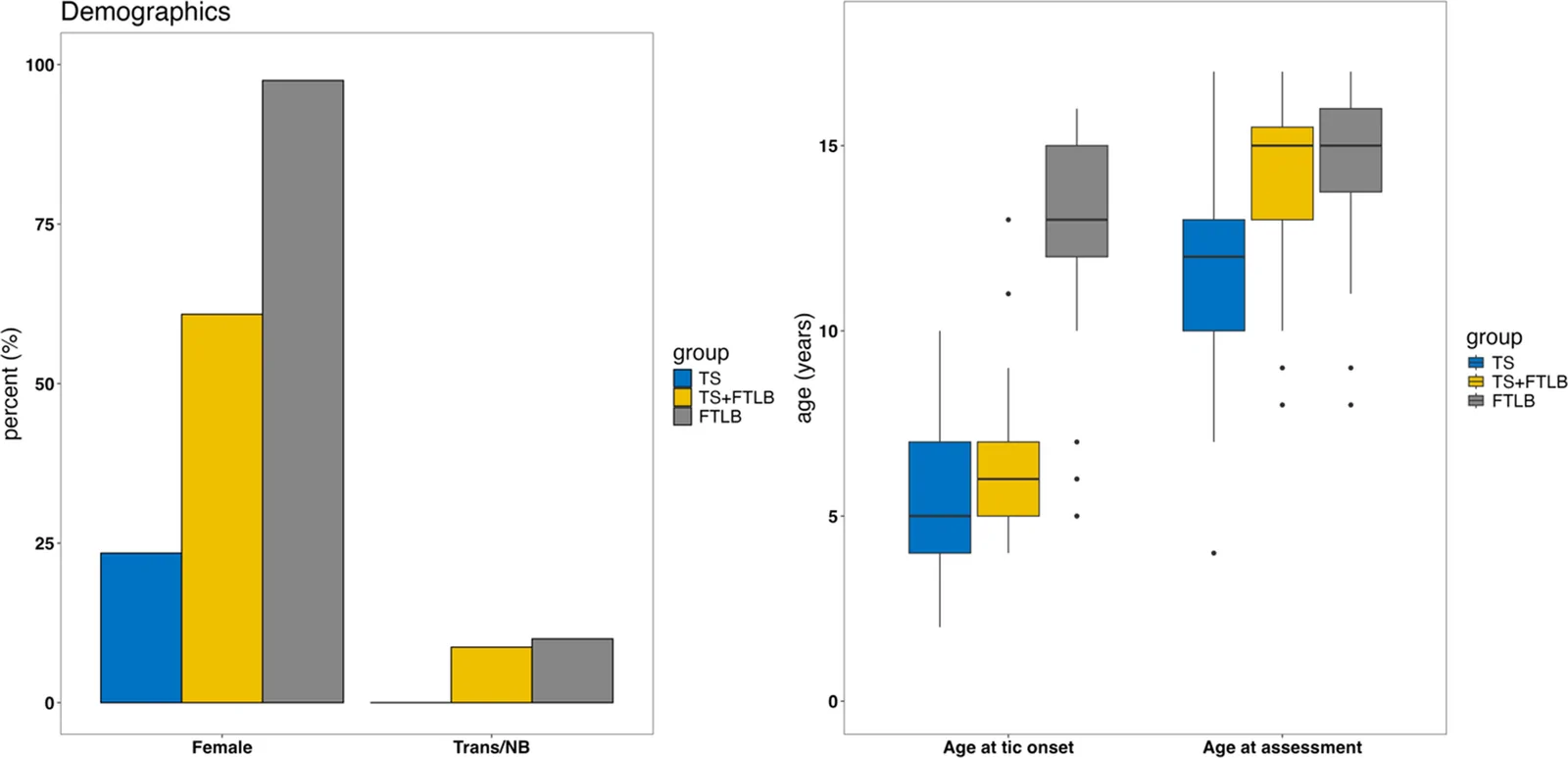
Bar chart and boxplots showing demographics by group

In line with international reporting [17], FTLB cases were significantly more likely to be female at birth, with later tic onset (Fig. 1; Table 1) and include the highest rate amongst the study groups of identification with being transgender/ nonbinary. Demographically, the FTLB + TS group shared features of both TS and FTLB.

### Neurodevelopmental and psychiatric co-morbidities

Table 1 shows findings of intergroup comparisons of the rates of co-occurring neurodevelopmental and psychiatric conditions. A psychiatric co-morbidity is significantly more common in FTLB groups (occurring in 78% or more of cases), mainly anxiety. Additional TS did not significantly increase likelihood of anxiety in FTLB cases. Depression is most common in FTLB, occurring in 25% of cases. The rate of depression in the FTLB + TS group fell between that of FTLB and TS groups.

**Table 1:** Table showing comparisons between TS, FTLB and FTLB + TS groups in relation to demographics, psychiatric and neurodevelopmental histories and family histories

Characteristic	Group			TS vs. TS+FTLB			TS+FTLB vs. FTLB			TS vs. FTLB		
	TS, *N* = 64^1^	TS+FTLB, *N* = 23^1^	**FTLB**, *N* = 40^1^	OR	95% CI	*p*-value	OR	95% CI	*p*-value	OR	95% CI	*p*-value
Female	15 (23%)	14 (61%)	39 (98%)	5.08	1.87, 14.6	**0.002**	0.04	0.00, 0.24	**0.003**	127	24.4, 2,357	**< 0.001**
Age at assessment	12.00 (10.00, 13.00)	15.00 (13.00, 16.00)	15.00 (13.50, 16.00)	1.42	1.15, 1.81	**0.002**	0.85	0.66, 1.08	0.2	1.71	1.38, 2.21	**< 0.001**
Age at tic onset	5.0 (4.0, 7.0)	6.0 (5.0, 7.0)	13.0 (12.0, 15.0)	1.25	0.99, 1.60	0.070	0.61	0.46, 0.75	**< 0.001**	1.94	1.56, 2.60	**< 0.001**
Minority ethnic background	29 (45%)	4 (17%)	18 (45%)	0.25	0.07, 0.77	0.023	0.26	0.07, 0.83	0.033	0.99	0.44, 2.19	> 0.9
Any ND dx	49 (77%)	22 (96%)	31 (78%)	6.73	1.24, 126	0.073	6.39	1.08, 122	0.089	1.05	0.42, 2.78	> 0.9
Autism	22 (34%)	17 (74%)	27 (68%)	5.41	1.95, 16.8	**0.002**	1.36	0.45, 4.51	0.6	3.97	1.74, 9.41	**0.001**
ADHD	28 (44%)	18 (78%)	14 (35%)	4.63	1.62, 15.4	**0.007**	6.69	2.16, 23.9	**0.002**	0.69	0.30, 1.55	0.4
spLD	15 (23%)	7 (30%)	5 (13%)	1.43	0.48, 4.06	0.5	3.06	0.85, 11.8	0.089	0.47	0.14, 1.33	0.2
OCD	8 (13%)	7 (30%)	4 (10%)	3.06	0.95, 9.86	0.058	3.94	1.04, 16.9	**0.049**	0.78	0.20, 2.66	0.7
Any psych dx	33 (52%)	18 (78%)	33 (83%)	3.38	1.19, 11.2	**0.031**	0.76	0.21, 2.90	0.7	4.43	1.79, 12.2	**0.002**
Dep	5 (7.8%)	4 (17%)	10 (25%)	2.48	0.57, 10.3	0.2	0.63	0.16, 2.19	0.5	3.93	1.28, 13.6	**0.021**
Anx	32 (50%)	18 (78%)	28 (70%)	3.60	1.26, 12.0	**0.023**	1.54	0.48, 5.52	0.5	2.33	1.03, 5.51	**0.047**
FHx of any ND dx	46 (72%)	12 (52%)	20 (50%)	0.43	0.16, 1.15	0.090	1.09	0.39, 3.08	0.9	0.39	0.17, 0.89	**0.026**
FHx Autism	29 (45%)	4 (17%)	9 (23%)	0.25	0.07, 0.77	**0.023**	0.73	0.18, 2.57	0.6	0.35	0.14, 0.83	**0.021**
FHx ADHD	25 (39%)	8 (35%)	9 (23%)	0.83	0.30, 2.21	0.7	1.84	0.58, 5.78	0.3	0.45	0.18, 1.08	0.083
FHx of any Psych dx	34 (53%)	16 (70%)	22 (55%)	2.02	0.75, 5.86	0.2	1.87	0.65, 5.78	0.3	1.08	0.49, 2.40	0.9
FHx Dep	20 (31%)	9 (39%)	9 (23%)	1.41	0.51, 3.79	0.5	2.21	0.72, 6.90	0.2	0.64	0.25, 1.56	0.3
FHx Anx	18 (28%)	13 (57%)	17 (43%)	3.32	1.25, 9.15	**0.017**	1.76	0.63, 5.06	0.3	1.89	0.82, 4.37	0.13
FHx Tics	30 (47%)	8 (35%)	3 (7.5%)	0.60	0.22, 1.59	0.3	6.58	1.66, 33.3	**0.011**	0.09	0.02, 0.29	**< 0.001**

^1^n (%); Median (Q1, Q3)

Abbreviations: *CI* Confidence Interval, *OR*  Odds RatioSignificance at 0.05 level in bold type

Autism was highly prevalent in both FTLB groups (> 65%), with autism prevalence in FTLB groups being significantly higher than in TS group, despite prevalence of autism in TS itself being high (34%). There was no significant difference between autism rates between FTLB and FTLB + TS groups.

ADHD has an established association with TS and therefore it was unsurprising to observe high rates of ADHD in both TS groups, however, it was of interest that the FTLB + TS group had a significantly higher rate of ADHD than the TS group, and that ADHD rates in FTLB was also much elevated compared to reported population base rates. Both findings suggest that as well as ADHD being associated with TS, it may well be independently associated with FTLB.

### Family history

**Fig. 2:**
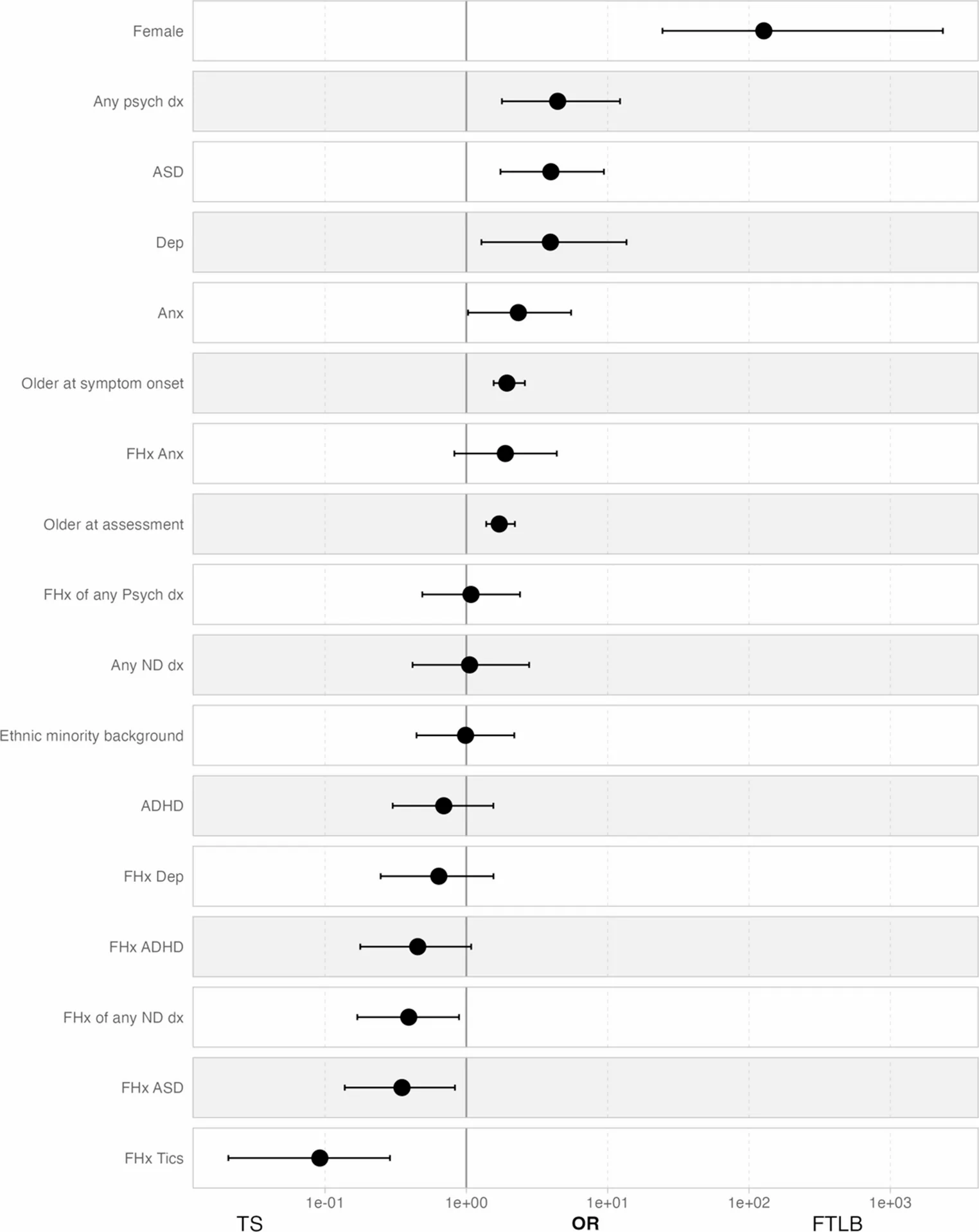
Forest plot showing odds ratios and 95% confidence intervals for the FTLB vs TS comparisons

Consistent with existing literature, family history of tics is more prominent in the TS group compared to FTLB groups. Interestingly, although co-occurring autism was common in FTLB, family history of autism is significantly higher in the TS group which appears counter-intuitive when considering autism is a highly genetic condition. In agreement with existing findings, family history of anxiety is more frequently found in FTLB, reaching significance in the FTLB + TS vs. TS comparison. Overall, our findings are consistent with existing literature differentiating TS from FTLB groups and Fig. 2 graphically represents their significant areas of difference (similar figures are available for the other group comparisons in the "supplementary data").

### Post-hoc exploration of ADHD in FTLB

The finding of higher-than-expected rates of ADHD in FTLB warranted additional exploration. 44% of young people in the TS group had ADHD which is in line with reported literature. This increases to 78% in cases of FTLB + TS suggesting ADHD is an additional risk for FTLB. This is also suggested by the rate of 35% of ADHD in young people with FTLB without TS. Those assigned female at birth accounted for 11% of those with ADHD in the TS group, but 55% in the FTLB + TS group and 100% in the FTLB group. This latter is expected given that FTLB is associated with female predominance.

From our dataset, we were able to readily identify when ADHD diagnosis was made. These were assigned to groups: before initial assessment at the tic service, at the initial assessment, or following the initial assessment. ADHD diagnosis within the tic service was provided if the assessing consultant had expertise in ADHD diagnosis. If not, where ADHD was suspected, referral was made back to local teams for ADHD assessment. Where ADHD diagnosis was made by local ADHD services and reported back to the tic service, for instance during the timeframe of tic service treatment, the ADHD diagnosis was recorded. 58% (*n* = 35) of all participants with ADHD had an existing diagnosis at tic assessment, 17% (*n* = 10) were diagnosed with ADHD in tic clinic and 25% (*n* = 15) were diagnosed by the patient’s local ADHD service. An additional 9 participants referred to local services for assessment of ADHD were either not diagnosed or were still awaiting diagnosis at time of discharge from the Tic Service and were not coded as having ADHD in this study.

**Fig. 3:**
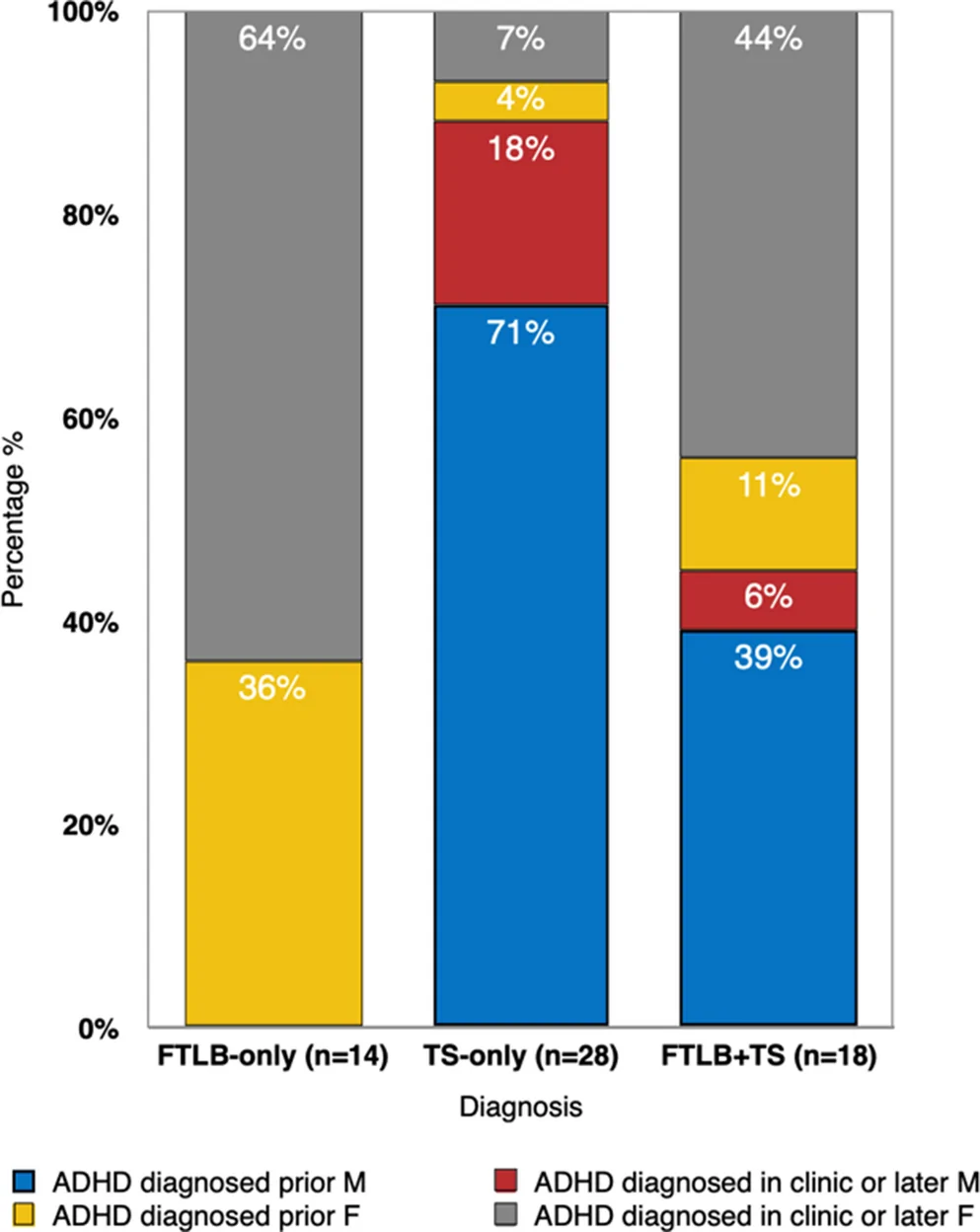
Sex distribution of cases with co-occurring ADHD across TS, FTLB and FTLB+TS groups, with further differentiation based on ADHD diagnosis being made before or at/ after clinical assessment for tics/ FTLB

ADHD was more readily identified in those with TS compared to FTLB groups with 75% of TS group having ADHD already diagnosed on first presentation to tic service, compared to 36% in the FTLB group and 50% in the FTLB + TS group. However, this is confounded by gender as 70% of ADHD was missed in girls at the time of initial presentation to the tic service, compared to only 18% in boys. Figure 3 shows the proportions of ADHD and time of diagnosis when the cohort is divided by sex assigned at birth and TS, FTLB and FTLB + TS groups.

## Discussion

### Mental health co-morbidity

We find results consistent with existing literature that FTLB can be differentiated from TS by gender, age of tic onset and presentation to services [17] and levels of anxiety and depression [4, 13]. As our sample consists of children diagnosed prior to June 2022, FTLB diagnoses were clinically made prior to establishment of the ESSTS consensus criteria for diagnosis of FTLB (which stipulates age 12 + and pre-existing anxiety and depression within diagnostic criteria) avoiding tautologous findings which have been highlighted as problematic [21]. Our findings lend support to the validity of the ESSTS diagnostic criteria. Presence of autism was identified as another differentiating factor between FTLB and TS, consistent with international FTLB findings and a growing research literature linking autism to functional symptoms [22, 23].

### Family history of mental health co-morbidity

In line with existing FTLB literature [19] and expectations given genetic predisposition to mental health conditions, family history of tics is markedly present in the tic group, whilst in FTLB where co-morbid anxiety is common, a family history of anxiety is more prominent. Interestingly, although the FTLB group had a significantly higher rate of autism than the TS group, family history of autism was significantly higher in the TS group compared to the FTLB groups. This was intriguing but may reflect the fact that autistic individuals that develop FTLB are typically female with mild autism and no intellectual disability. Historically only more marked and ‘classic’ presentations of autism were diagnosed, and family histories may not have captured milder phenotypes that may well have been present [24].

### ADHD in FTLB

ADHD was found to be highly prevalent in both participants with TS and FTLB, contrary to previous findings that ADHD may be more prevalent in organic tic disorders as compared to FLTB and used as a clinical differentiator [25]. TS is known to be frequently comorbid with ADHD [26], likely due to shared genetic risk factors between tic disorders and ADHD as shown by genetic polymorphism studies [27] and twin studies [28]. The prevalence of ADHD was higher in our study for both TS and FTLB participants compared to previous studies, including adult studies, where it ranged from 16% to 44% in organic tic disorders and 19–25% in FTLB [5, 9, 19]. The prevalence becomes similar if only those with a prior ADHD diagnosis are considered, which suggests ADHD may be frequently undetected if not systematically assessed for in this patient group. Notably, whereas those with TS were more likely to have a prior diagnosis of ADHD, the majority of those with FTLB had ADHD diagnosed during or following the tic clinic assessment. These disparities may reflect the gender profiles as FTLB cases are predominantly female. Girls are less likely to have been diagnosed with ADHD, which has been found in other tic cohorts [29]. This may be due to under recognition of symptom profiles in girls and system biases [30], making routine enquiry about ADHD symptoms in girls even more important.

As anxiety is a well-documented consequence of ADHD in females [30], it is possible that anxiety generated from undiagnosed/untreated ADHD is a contributory factor to FTLB. Future studies assessing this mechanism are required, as effective treatments are available for ADHD and this may be a preventative intervention for FTLB.

### FTLB + TS group

The small subgroup of individuals with both TS and FTLB could be differentiated from the FTLB subgroup by increased levels of ADHD and family history of tics, reflecting commonalities of the FTLB + TS group to both FTLB and TS. ADHD was most prevalent in those with both TS and FTLB. This likely reflects a cohort of young people with mixed neuropsychiatric needs which makes them especially vulnerable to FTLB.

### Limitations

Our study has several limitations. Given this was a pragmatic study, follow-up was opportunistic, meaning that outcomes for some participants who were referred for autism and ADHD assessments were not known. However, this would only underestimate prevalence as only those with a confirmed diagnosis were positively coded. The small sample size in our cohort excludes the use of multivariate analyses adjusting for confounders which would be ideal; however, our sample is larger than comparable studies [3, 5, 19] as the prevalence of FTLB is low. Replication of these findings in larger samples sufficiently powered to adjust for confounders is required, although this is likely only possible through international multi-centre cohorts over a longer period.

This clinical sample is from a national specialist centre so may not be generalisable to community services or the general population. As specialist centres tend to be referred more complex cases and those resistant to community treatment, there are likely higher rates of co-morbidity due to the specialist nature of the setting. There is also the well-established difficulty in distinguishing between organic tics and FTLB, including overlaps, which without an objective test affects all clinical studies on this topic [31] . No male patients were diagnosed with FTLB without TS which raises the possibility of biases. As this was a cross-sectional study, it was not possible to establish temporal relationship between e.g. anxiety/depression and FTLB or causality, and we recommend further research into mechanistic models.

### Summary

Our findings support existing literature linking increased levels of anxiety and depression to FTLB compared to TS, supporting ESSTS diagnostic criteria for FTLB. FTLB is also more likely to be associated with family history of anxiety, in line with extant studies. Improving identification, diagnosis, and treatment of ADHD, particularly in girls, is advised. Future work to assess whether undiagnosed ADHD may predispose to FTLB and potential mechanisms should be considered, which may also have implications for FND and research into ADHD in FND should also be a consideration.

## Supplementary Information

Below is the link to the supplementary material.

Supplementary Material 1 (DOCX 723 KB)

## Data Availability

All non-confidential raw data generated in this study are available from the authors on appropriate request.

## References

[1] Mittal SO (2020) Tics and Tourette’s syndrome. Drugs Context 9:2019–12–2. 10.7573/dic.2019-12-2 PMC7111125 32273897

[2] Müller-Vahl KR, Pisarenko A, Jakubovski E, Fremer C (2022) Stop that! It’s not Tourette’s but a new type of mass sociogenic illness. Brain 145(2):475–480 10.1093/brain/awab316 PMC9014744 34424292

[3] Buts S, Duncan M, Owen T, Martino D, Pringsheim T, Byrne E et al (2022) Paediatric tic-like presentations during the COVID-19 pandemic. Arch Dis Child 107(3):e17 10.1136/archdischild-2021-323002 34824091

[4] Han VX, Kozlowska K, Kothur K, Lorentzos M, Wong WK, Mohammad SS et al (2022) Rapid onset functional tic-like behaviours in children and adolescents during COVID‐19: clinical features, assessment and biopsychosocial treatment approach. J Paediatr Child Health 58(7):1181–1187 10.1111/jpc.15932 PMC9115185 35247213

[5] Pringsheim T, Ganos C, McGuire JF, Hedderly T, Woods D, Gilbert DL et al (2021) Rapid onset functional tic-like behaviors in young females during the COVID-19 pandemic. Mov Disord 36(12):2707–2713 10.1002/mds.28778 PMC8441698 34387394

[6] Daly M, Robinson E (2021) Longitudinal changes in psychological distress in the UK from 2019 to September 2020 during the COVID-19 pandemic: Evidence from a large nationally representative study. Psychiatry Res 300:113920 10.1016/j.psychres.2021.113920 PMC9755113 33882397

[7] Drouin M, McDaniel BT, Pater J, Toscos T (2020) How parents and their children used social media and technology at the beginning of the COVID-19 pandemic and associations with anxiety. Cyberpsychol Behav Soc Netw 23(11):727–736 10.1089/cyber.2020.0284 32726144

[8] Hull M, Parnes M (2021) Tics and TikTok: functional tics spread through social media. Mov Disord Clin Pract 8(8):1248–1252 10.1002/mdc3.13267 PMC8564820 34765689

[9] Pringsheim T, Martino D (2021) Rapid onset of functional tic-like behaviours in young adults during the COVID-19 pandemic. Eur J Neurol 28(11):3805–3808 10.1111/ene.15034 PMC8444872 34293224

[10] Rigas A, Mainka T, Pringsheim T, Münchau A, Malaty I, Worbe Y, Cavanna AE, Lees AJ, Lang AE, Martino D, Ganos C (2023) Distinguishing functional from primary tics: a study of expert video assessments. J Neurol Neurosurg Psychiatry 94(9):751–756. 10.1136/jnnp-2022-330822 PMC10447361 37169545

[11] Benbadis SR, Agrawal V, Tatum (2001) WO 4th. How many patients with psychogenic nonepileptic seizures also have epilepsy? Neurology 57(5):915–917 10.1212/wnl.57.5.915 11552032

[12] Amorelli G, Martino D, Pringsheim T (2022) Rapid onset functional tic-like disorder outbreak: a challenging differential diagnosis in the COVID-19 Pandemic. J Can Acad Child Adolesc Psychiatry 31(3):144–151 PMC9275373 35919901

[13] Cavanna AE, Caimi V, Capriolo E, Marinoni M, Arienti G, Riva A, Nacinovich R, Seri S (2025) Neurodevelopmental tics with co-morbid functional tic-like behaviors: diagnostic challenges of a complex tourette syndrome phenotype. Brain Sci 15(5):435. 10.3390/brainsci15050435 PMC12110104 40426606

[14] Howlett M, Martino D, Nilles C, Pringsheim T (2022) Prognosis of rapid onset functional tic-like behaviors: prospective follow-up over 6 months. Brain Behav 12(6):e2606. 10.1002/brb3.2606 PMC9226799 35593445

[15] Duncan M, Pearman Z, Harrold K, Warren A, Evans S, McAllister E, Heyman I, Shavel-Jessop S, Murphy T, Liang H (2024) Evaluation of a psychoeducation group for children presenting with functional tic-like behaviours. Clin Child Psychol Psychiatry 29(3):1011–1025 10.1177/13591045241237829 38476087

[16] Pringsheim T, Ganos C, Nilles C, Cavanna AE, Gilbert DL, Greenberg E et al (2023) European society for the study of tourette syndrome 2022 criteria for clinical diagnosis of functional tic-like behaviours: international consensus from experts in tic disorders. Eur J Neurol 30(4):902–910 10.1111/ene.15672 36587367

[17] Martino D, Hedderly T, Murphy T, Müller-Vahl KR, Dale RC, Gilbert DL et al (2023) The spectrum of functional tic-like behaviours: data from an international registry. Eur J Neurol 30(2):334–343 10.1111/ene.15611 36282623

[18] Hansen AS, Rask CU, Christensen AE, Rodrigo-Domingo M, Christensen J, Nielsen RE (2021) Psychiatric disorders in children and adolescents with psychogenic nonepileptic seizures. Neurology 97(5):e464–e475. 10.1212/WNL.0000000000012270 34031196

[19] Andersen K, Jensen I, Okkels KB, Skov L, Debes NM (2023) Clarifying the differences between patients with organic tics and functional tic-like behaviors. Healthc (Basel) 11(10):1481 10.3390/healthcare11101481 PMC10217976 37239767

[20] McFarlane FA, Allcott-Watson H, Hadji-Michael M, McAllister E, Stark D, Reilly C, Bennett SD, McWillliams A, Heyman I (2019) Cognitive-behavioural treatment of functional neurological symptoms (conversion disorder) in children and adolescents: a case series. Eur J Paediatr Neurol 23(2):317–328. 10.1016/j.ejpn.2018.12.002 30594458

[21] Andersen K, Cavanna AE, Szejko N, Müller-Vahl KR, Hedderly T, Skov L (2024) Mol Debes N. A critical examination of the clinical diagnosis of functional tic-like behaviors. Mov Disord Clin Pract 11(9):1065–1071 10.1002/mdc3.14150 PMC11452787 38940477

[22] González-Herrero B, Morgante F, Pagonabarraga J, Stanton B, Edwards MJ (2022) Autism spectrum disorder may be highly prevalent in people with functional neurological disorders. J Clin Med 12(1):299 10.3390/jcm12010299 PMC9821674 36615098

[23] Tamilson B, Poole N, Agrawal N (2024) The co-occurrence of functional neurological disorder and autism spectrum disorder: a systematic literature review and meta-analysis. Cogn Neuropsychiatry 29(6):358–385 10.1080/13546805.2025.2452259 39888594

[24] Goldman S, Opinion (2013) Sex, gender and the diagnosis of autism - a biosocial view of the male preponderance. Res Autism Spectr Disord 7(6):675–679. 10.1016/j.rasd.2013.02.006 PMC3655776 23687516

[25] Cavanna AE, Purpura G, Riva A, Nacinovich R, Seri S (2023) Neurodevelopmental versus functional tics: a controlled study. J Neurol Sci 451:120725. 10.1016/j.jns.2023.120725 37421881

[26] Freeman RD, Fast DG, Burd L, Kerbeshian J, Robertson MM, Sandor P (2000) An international perspective on Tourette syndrome: selected findings from 3500 individuals in 22 countries. Dev Med Child Neurol 42:436–447 10.1017/s0012162200000839 10972415

[27] Gadow KD, Roohi J, DeVincent CJ, Hatchwell E (2008) Association of ADHD, tics, and anxiety with dopamine transporter (DAT1) genotype in autism spectrum disorder. J Child Psychol Psychiatry 49(12):1331–1338 10.1111/j.1469-7610.2008.01952.x PMC4349425 19120712

[28] Lichtenstein P, Carlström E, Råstam M, Gillberg C, Anckarsäter H (2010) The genetics of autism spectrum disorders and related neuropsychiatric disorders in childhood. Am J Psychiatry 167(11):1357–1363 10.1176/appi.ajp.2010.10020223 20686188

[29] Girgis J, Martino D, Pringsheim T (2022) Influence of sex on tic severity and psychiatric comorbidity profile in patients with pediatric tic disorder. Dev Med Child Neurol 64(4):488–494 10.1111/dmcn.15088 34672366

[30] Young S, Adamo N, Ásgeirsdóttir BB, Branney P, Beckett M, Colley W et al (2020) Females with ADHD: an expert consensus statement taking a lifespan approach providing guidance for the identification and treatment of attention-deficit/ hyperactivity disorder in girls and women. BMC Psychiatry 20(1):404 10.1186/s12888-020-02707-9 PMC7422602 32787804

[31] Ganos C, Martino D, Espay AJ, Lang AE, Bhatia KP, Edwards MJ (2019) Tics and functional tic-like movements: can we tell them. apart? Neurol 93(17):750–758 10.1212/WNL.0000000000008372 31551261

