# Supplementary material for: Differentiating tics and functional tic-like behaviours in children and young people: personal and family mental health profiles and their clinical implications
Source: Eur Child Adolesc Psychiatry. 2026 May 26;35(9):2939–47. doi: 10.1007/s00787-026-03067-7 (PMC13695623; doi:10.1007/s00787-026-03067-7)
Supplement: Supplementary file 1 [file 787_2026_3067_MOESM1_ESM.docx]

**Supplementary Figures:**

Figure S2. Forest plot showing Odds Ratios and 95% Confidence Intervals for the TS vs TS+FTLB comparisons.

Figure S3. Forest plot showing Odds Ratios and 95% Confidence Intervals for the FTLB vs TS+FTLB comparisons.
